# Brain stimulation over dorsomedial prefrontal cortex modulates effort-based decision making

**DOI:** 10.3758/s13415-022-01021-z

**Published:** 2022-06-21

**Authors:** Alexander Soutschek, Lidiia Nadporozhskaia, Patricia Christian

**Affiliations:** 1 Department for Psychology, Ludwig Maximilian University Munich, Leopoldstr. 13, 80802 Munich, Germany; 2 Department of Biology, Graduate School for Systemic Neurosciences, Ludwig Maximilian University Munich, Munich, Germany; 3 Institute of Molecular and Clinical Ophthalmology, University of Basel, Basel, Switzerland

**Keywords:** Mental effort, Oscillations, Decision making, Working memory, Transcranial alternating current stimulation

## Abstract

Deciding whether to engage in strenuous mental activities requires trading-off the potential benefits against the costs of mental effort, but it is unknown which brain rhythms are causally involved in such cost-benefit calculations. We show that brain stimulation targeting midfrontal theta oscillations increases the engagement in goal-directed mental effort. Participants received transcranial alternating current stimulation over dorsomedial prefrontal cortex while deciding whether they are willing to perform a demanding working memory task for monetary rewards. Midfrontal theta tACS increased the willingness to exert mental effort for rewards while leaving working memory performance unchanged. Computational modelling using a hierarchical Bayesian drift diffusion model suggests that theta tACS shifts the starting bias before evidence accumulation towards high reward-high effort options without affecting the velocity of the evidence accumulation process. Our findings suggest that the motivation to engage in goal-directed mental effort can be increased via midfrontal tACS.

## Introduction

When deciding whether to read a scientific article, we need to trade-off the benefits of reading a potentially interesting article against the mental effort required to read it. Because mental effort is perceived as costly, humans should engage in mentally demanding activities only if the potential benefits outweigh the costs of exerting effort (Shenhav et al., 2017; Soutschek & Tobler, 2018). Exertion of goal-directed mental effort was linked to midfrontal theta oscillations (Cooper et al., 2019; McFerren et al., 2021; Pastötter et al., 2013). Midfrontal theta oscillations are thought to be generated by rhythmic neural activity in dorsomedial prefrontal (dmPFC) and dorsal anterior cingulate cortex (dACC) (Holroyd & Umemoto, 2016). Previous imaging studies suggest that these regions to be engaged in cost-benefit weighting (Bonnelle et al., 2016; Klein-Flugge et al., 2016; Vassena et al., 2017; Westbrook et al., 2019). Yet, causal evidence for the contribution of midfrontal theta rhythms to effort-based choice is missing so far. Previous studies using transcranial alternating current stimulation (tACS) showed that theta tACS over dmPFC energizes cognitive control processes (Fusco et al., 2018; van Driel et al., 2015). Because the recruitment of cognitive control is thought to rely on a rational decision process weighing the potential benefits of exerting control against its costs (Shenhav et al., 2013; Shenhav et al., 2017), these findings are consistent with a role of midfrontal theta for motivating mental effort. However, no study so far has investigated whether midfrontal theta oscillations causally implement cost-benefit trade-offs.

We applied tACS to test the causal involvement of midfrontal theta oscillations in effort-based decision making. While receiving tACS over dmPFC, participants decided whether to engage in a demanding working memory (N-back) task for monetary rewards (Westbrook et al., 2013; Westbrook et al., 2020). Based on theoretical accounts on dmPFC involvement in cost-benefit weighting, we hypothesized that entrainment of midfrontal theta oscillations increases the willingness to engage in rewarded effort. To obtain a more fine-grained picture of the role of midfrontal oscillations for effort-based choice, we analyzed tACS effects on subcomponents of the choice process in a drift diffusion model. From the perspective of process models, the role of midfrontal oscillations in effort-based decisions could be explained via different mechanisms: First, if midfrontal oscillations are involved in weighing the benefits against the costs of actions (Shenhav et al., 2013; Shenhav et al., 2017), then midfrontal tACS should change the influences of rewards and effort costs on the speed of evidence accumulation. In this case, strengthening midfrontal oscillations would not promote choices of high effort options per se, but rather improve the trade-off process weighing reward values against subjective effort costs (which can lead to either acceptance or rejection of high effort options depending on whether a reward is worth the effort). Alternatively, in line with animal studies showing that ACC lesions reduce the willingness to engage in mental effort (Hosking et al., 2014; Walton et al., 2009), entrainment of midfrontal theta oscillations may shift the starting point of the accumulation process closer towards high effort-high reward options. Lastly, to rule out that the stimulation changed decision making by affecting the capacity to exert mental effort rather than motivational processes, we controlled for tACS effects on working memory performance. If midfrontal theta oscillations improve the motivation to work for rewards without affecting the capacity to exert mental effort, we expected to observe effects of tACS only on the decision task, not on the (un-incentivized) working memory task.

## Materials and methods

### Participants

Thirty-five, healthy, young volunteers (16 females; M_age_ = 25.4, years, range = 19-33) were recruited from the student population in Munich. A power analysis assuming an effect size of Cohen’s d = 0.54 observed in a previous tACS study from our group (Soutschek, Moisa, et al., 2021b) suggests that 29 participants are sufficient to detect significant effects (*p* = 0.05, two-tailed) with a power of 80%. One participant erroneously performed the 1-back instead of the 2-back task during tACS (see below); the 2-back data of this participant were therefore excluded from the analysis. All volunteers were screened for possible contraindications of tDCS and gave voluntary informed consent prior to participation. They received a compensation of 10 euro/hour and additionally a bonus depending on their choices (see below). The study was approved by a local ethics committee.

### Stimuli and task design

Participants performed two tasks: an N-back working memory task and an effort-based decision task. In the N-back task, we presented participants a stream of letters; each letter was presented for 1.5 s (intertrial interval: 1.5 s). The task was to decide via keypress whether the currently presented letter was identical with the letter presented N trials before. For illustration purpose, participants performed example blocks (12 trials each) for the 1-back, 2-back, 3-back, and 4-back conditions before receiving instructions for the decision task in order to familiarize them with the subjective effort demands. During stimulation, participants performed only the 2-back condition, because this difficulty level was shown to be sensitive to stimulation effects (Mottaghy et al., 2003; Sandrini et al., 2008; Soutschek & Tobler, 2020). This allowed us to assess whether tACS over dmPFC influences the capacity to exert mental effort.

**Fig. 1:**
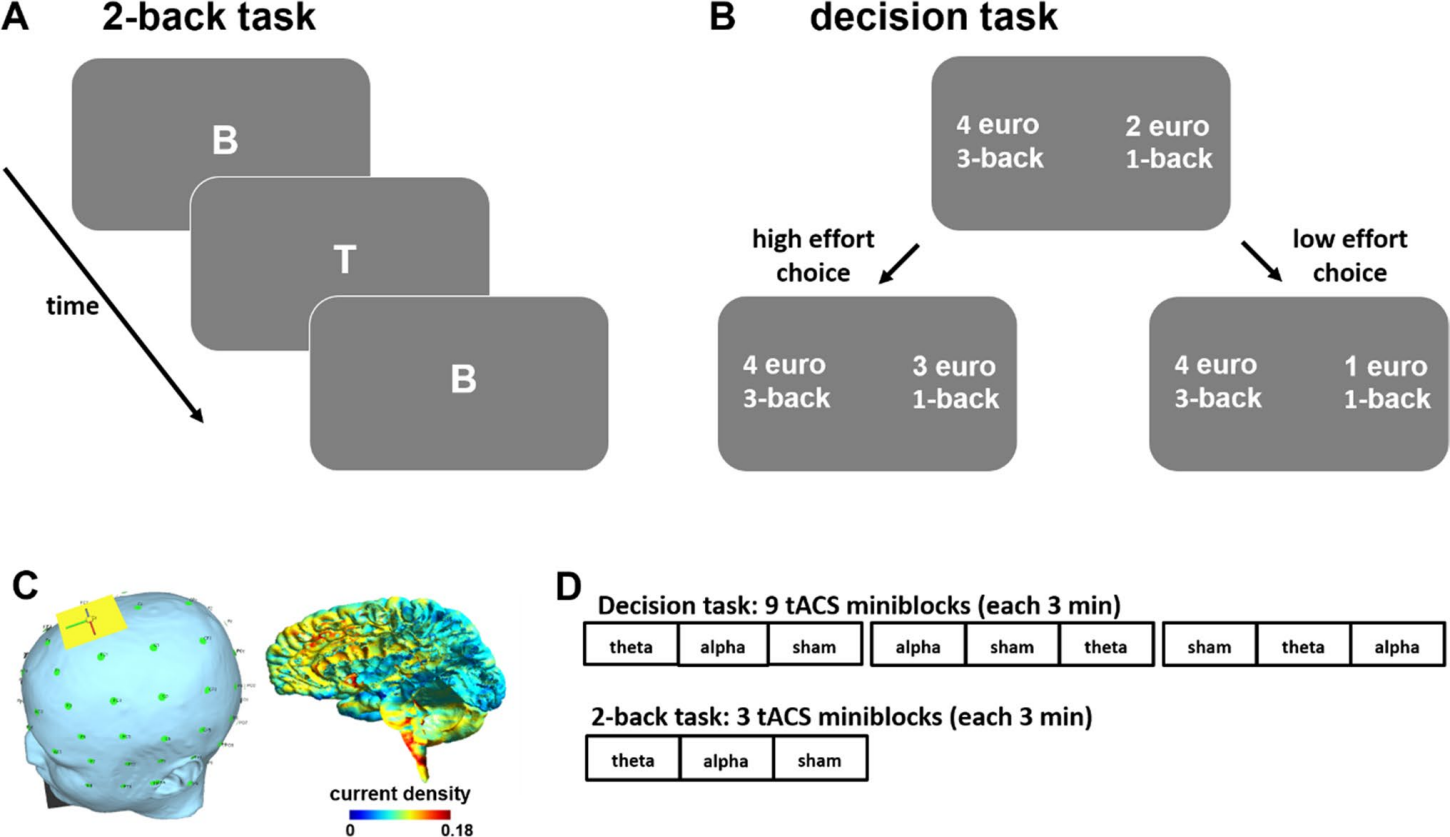
**Task design and experimental procedures**. **a** While receiving tACS, participants performed a 2-back working memory task to control for potential stimulation effects on the capacity to exert mental effort. **b** In the effort-based decision task, participants decided between a low effort option and a high effort option. We used a titration procedure where the reward magnitude of the low effort option was adjusted depending on participants’ choices. **c** We administered theta, alpha, and sham tACS over the dmPFC (return electrode was under the chin) with a current strength of 1.5 mA peak-to-peak. Current density simulations with Simnibs suggest that with this setup the current density is highest in the dmPFC and dACC. **d** Participants performed the effort-based decision task and the 2-back working memory task in miniblocks under either theta, sham, or alpha tACS

In the effort-based decision task (Westbrook et al., 2013; Westbrook et al., 2020), participants decided on each trial whether they prefer a low reward-low effort option or a high reward-high effort option (Fig. 1a). For the high reward-high effort option, the amount of the monetary reward was fixed to either 2 or 4 euro, and the required effort ranged from 2-back to 4-back. For the low reward-low effort option, the effort level varied from 1-back to 3-back and the monetary offer was dynamically adjusted after each choice using a titration method. On the first trial for each low effort-high effort combination, the amount of the low effort option was set to half the amount of the high effort option (i.e., either 1 or 2 euro). Then, the amount of the low effort option was adjusted after each choice such that the difficulty of the following choice was maximized. For example, if a participant first chose the high effort option when deciding between 2 euro for low effort and 4 euro for high effort, the amount of the low effort option was adjusted to 3 euro in the next trial. If the participant then decided for the low effort option, the amount of the low effort option was adjusted to 2.5 euro. Participants performed five consecutive choices for each combination of low effort and high effort demands, which allowed us to estimate the subjective values of the reward options with a precision of 0.0625 euro.

At the end of the experiment, we randomly selected and implemented one choice of each participant. In case participants had chosen the high effort option, they had to perform the corresponding level of the N-back task for 2 min to obtain the reward. Following previous procedures, we informed participants that they would win the bonus as long as they worked hard for it (Soutschek & Tobler, 2020; Westbrook et al., 2013). This ensures that choices are guided by participants’ evaluation of mental effort requirements rather than by the perceived risk of not obtaining the bonus despite strong task engagement.

### tACS protocol

We applied tACS using an 8-channel tDCS stimulator (DC-stimulator MC, neuroConn, Ilmenau, Germany). To modulate midfrontal theta oscillations, we placed a 5×5 cm electrode over electrode position FCz according to the international 10-20 system as well as a 5×5 cm reference electrode under the chin (Fig. 1b). The electrode position FCz was determined by measuring 40% of the individual nasion-inion distance from the nasion. The electrodes were fixed to the participants’ heads by rubber straps. We stimulated participants in the theta band (5 Hz) and alpha band (9.7 Hz) frequency with a current strength of 1.5 mA (peak-to-peak). We chose an alpha frequency of 9.7 Hz as control condition in order to follow the procedures of a previous tACS study on the role of midfrontal theta for cognitive control (van Driel et al., 2015). Stimulation conditions were matched with regard to stimulation-induced perceived discomfort, both *Z* < 1, both *p* > 0.43, or flickering, both *Z* < 1.61, both *p* > 0.10.

To assess which brain regions were affected by the electrode setup, we performed current flow simulations with the Simnibs 3.2 toolbox (Saturnino et al., 2019). Based on previous neuroimaging studies on mental effort (Chong et al., 2017; Schmidt et al., 2012), we defined spherical regions-of-interests (ROIs; 10 mm diameter) for regions involved in effort-based choice (dACC: x = −4, y = 22, z = 44; dlPFC: x = −44, y = 26, z = 26; striatum: x = −14, y = 10, z = 18; insula: x = 34, y = 22, z = 4; intraparietal sulcus (IPS): x = 36, y = −44, z = 38). We then extracted the local field strengths from these ROIs. The electrical field was strongest in the dACC ROI (mean electrical field (EF) = 0.09 V/m), followed by the dlPFC (mean EF = 0.08 V/m), the insula (mean EF = 0.07 V/m), the striatum (mean EF = 0.06 V/m), and the IPS (mean EF = 0.06 V/m). While the electrical current was relatively strong also in the dlPFC, we note that we control for stimulation effects on dlPFC by assessing whether the current tACS protocol affected working memory performance in the 2-back task. As robustness check, we also simulated the electrical field under the assumption that the dmPFC electrode slipped 5% of the nasion-inion distance either in the anterior or the posterior direction, but this did not substantially change field strengths in the ROIs.

### Procedure

Participants performed the decision task and the 2-back task (in counterbalanced order) within one experimental session (Fig. 1c). We administered the decision task in 9 miniblocks (3 per tACS condition) of 20 trials and the 2-back task in 3 miniblocks (1 per tACS condition) of 40 trials. We counterbalanced the order of tACS conditions using Latin square methods.

Each miniblock started with a ramp-up period of 5 s for the tACS current. In the sham condition, the current was ramped-down directly after the ramp-up phase. In 15 s after the start of tACS, participants performed the decision and the 2-back tasks for 115 s. After task performance, the current was ramped-down over a period of 5 s. Starting with the ramp-down phase, participants had to indicate the perceived aversiveness of the stimulation and the tACS-induced flickering on rating scales from 0 to 20. The next block started after a stimulation-free interval of 40 s.

### Data analysis

For the effort-based decision task, we conducted model-based and model-free analyses. For the model-free analysis, we computed a Bayesian mixed general linear model (MGLM) using the brms package in R (Bürkner, 2017). The titration method used in the decision task allowed us to determine the relative subjective value of the high effort-option relative to demands of the low effort-option (Westbrook et al., 2020). We regressed the relative subjective values for each combination of low effort and high effort demands on fixed-effects predictors for tACS (tACS_theta-sham_ and tACS_alpha-sham_), the difference between high and low effort requirements (Effort_diff_), the reward magnitude of the high effort option (Reward_high_), and the interaction terms (MGLM-1). All predictors were also modelled as random slopes in addition to participant-specific random intercepts. To control for potential confounding effects of tACS-induced discomfort or flickering as well as of the order in which the experimental tasks and tACS conditions were administered, we added these variables as predictors of no interest (note that none of these control variables showed a significant result). We used noninformative flat priors (default in brms) and estimated the posterior distributions of the parameter estimates by computing two chains with 4,000 samples (burn-in: 2,000 samples). For all parameter estimates, $\hat{R}$ was below 1.01, indicating model convergence. The significance of group-level effects was assessed using the 95% highest density interval (HDI_95%_).

In addition, we analyzed tACS effects on effort-based choices with Bayesian drift diffusion modelling (DDM) using the JAGS software package (Plummer, 2003). JAGS utilizes Markov Chain Monte Carlo sampling to estimate parameters of a drift diffusion model (drift rate v, boundary a, bias z, and nondecision time t) via the Wiener module (Wabersich & Vandekerckhove, 2014). The lower and upper boundaries (decision threshold) were associated with choices of the low and high effort options, respectively. In DDM_attribute_, we assumed that the drift rate v depends on the comparisons of reward magnitudes and effort costs between the low effort and high effort options (Soutschek et al., 2022; Westbrook et al., 2020):

1 $$\upnu =\left({\upbeta}_{\mathrm{reward}-\mathrm{sham}}+{\upbeta}_{\mathrm{reward}-\mathrm{theta}}\times {\mathrm{tACS}}_{\mathrm{theta}}+{\upbeta}_{\mathrm{reward}-\mathrm{alpha}}\times {\mathrm{tACS}}_{\mathrm{alpha}}\right)\times {\mathrm{Reward}}_{\mathrm{diff}}\hbox{--} \left({\upbeta}_{\mathrm{effort}-\mathrm{sham}}+{\upbeta}_{\mathrm{effort}-\mathrm{theta}}\times {\mathrm{tACS}}_{\mathrm{theta}}+{\upbeta}_{\mathrm{effort}-\mathrm{alpha}}\times {\mathrm{tACS}}_{\mathrm{alpha}}\right)\times {\mathrm{Effort}}_{\mathrm{diff}}$$

Reward_diff_ and Effort_diff_ indicate the difference between the reward magnitudes and effort requirements of the high and low effort options, respectively. tACS_theta_ and tACS_alpha_ were dummy-coded variables that were set to 1 for theta tACS and alpha tACS, respectively, and to 0 for all other conditions. This allowed us to model tACS effects on DDM parameters relative to the baseline sham condition on a within-subject level, in analogy to the model-free MGLM analysis. To investigate stimulation effects on DDM parameters, we modelled individual and group-level effects of theta and alpha tACS on all DDM parameters in a hierarchical Bayesian approach. We excluded the trials with the 5% fastest and slowest response times to reduce the impact of outliers on parameter estimation. We used non-informative uniform priors over numerically plausible parameter ranges and estimated parameters by computing two chains with 50,000 samples (burning = 40,000, thinning = 5). Again, $\hat{R}$ was below 1.01 for all parameter estimates.

Note that DDM_attribute_ assumes that participants decide between the reward options by making attribute-wise comparisons between the reward magnitudes and the associated N-back demands of the low and the high effort options. In contrast, alternative accounts posit that decision makers compare the discounted subjective values of the reward options (“effort discounting”). In the literature, there is no agreement on the precise shape of the discount function (Bialaszek et al., 2017; Chong et al., 2017; Schmidt et al., 2012). We therefore computed three further DDMs assuming that subjective effort costs are computed via either hyperbolic, parabolic, or linear discount functions, following previous procedures (Soutschek et al., 2020; Soutschek & Tobler, 2020):

2 $$\mathrm{SV}=\mathrm{Reward}/\left(1+{\upbeta}_{\mathrm{discount}}\times \mathrm{Effort}\right)\ \left(\mathrm{hyperbolic}\right)$$

3 $$\mathrm{SV}=\mathrm{Reward}\hbox{--} {\upbeta}_{\mathrm{discount}}\times {\mathrm{Effort}}^2\ \left(\mathrm{parabolic}\right)$$

4 $$\mathrm{SV}=\mathrm{Reward}\hbox{--} {\upbeta}_{\mathrm{discount}}\times \mathrm{Effort}\ \left(\mathrm{linear}\right)$$

We assumed that evidence for the reward options is accumulated by comparing the subjective values of the high effort and the low effort option:

5 $$\mathrm{v}={\mathrm{SV}}_{\mathrm{high}\ \mathrm{effort}}\hbox{--} {\mathrm{SV}}_{\mathrm{low}\ \mathrm{effort}}$$

We estimated these model parameters (as well as the effects of theta and alpha tACS on them relative to sham) in three separate DDMs in the same way as for DDM_attribute_. We compared the model fit between these models with the deviance information criterion (DIC).

Finally, we analyzed tACS effects on performance in the 2-back task. Again, we computed Bayesian MGLMs with the brms package in order to assess whether theta or alpha tACS, relative to sham tACS, affected mean reaction times (MGLM-2) on hit trials or d’ (z-transformed hit rate minus false alarms, which represents a measure of 2-back target detection performance, see Soutschek and Tobler (2020); MGLM-3). As for the analysis of the decision task, we controlled for potential influences of tACS-induced discomfort and flicker as well as of the order of task and stimulation conditions.

## Results

### Midfrontal theta tACS increases motivation to engage in mental effort

**Fig. 2:**
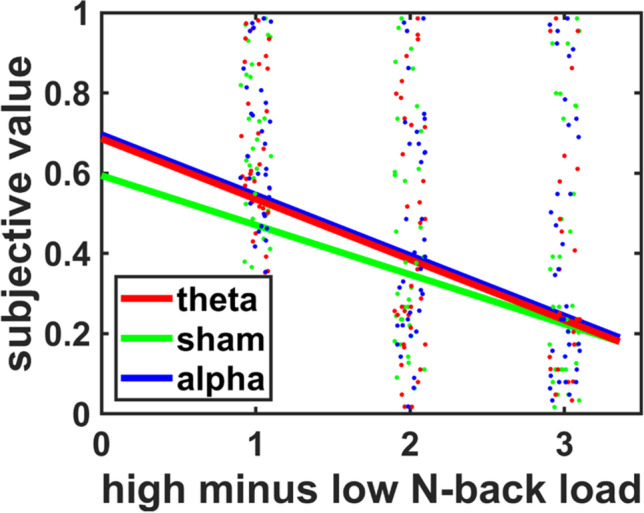
**Theta and alpha tACS increased the relative subjective value of a high effort option relative to sham tDCS.** The x-axis depicts the difference between the N-back levels of the high effort option minus the low effort option. Regression lines are based on the Bayesian MGLM results; black dots indicate individual data points

We first tested the hypothesized impact of midfrontal theta tACS on effort-based choice. We regressed the subjective values of the high effort options on predictors for tACS_theta-sham_, tACS_alpha-sham_, the difference between high and low effort demands (Effort_diff_), and the reward magnitude of the high effort option (Reward_high_). As expected, subjective values of high effort options decreased with increasing effort demands, HDI_mean_ = −0.06, HDI_95%_ = [−0.09; −0.03]. Consistent with our hypothesis, theta tACS significantly increased subjective values of high effort options relative to sham tACS, HDI_mean_ = 0.04, HDI_95%_ = [0.01; 0.07] (Fig. 2; Table 1), but we observed a significant main effect also for alpha tACS, HDI_mean_ = 0.06, HDI_95%_ = [0.03; 0.10]. Thus, both midfrontal theta and alpha tACS enhanced the willingness to exert mental effort for rewards.

**Table 1:** Results for Bayesian MGLM-1. Standard errors of the mean of the posterior distributions are in brackets

	Mean	2.5%	97.5%
Intercept	0.48 (0.11)	0.27	0.69
tACS_theta_	0.04 (0.02)	0.01	0.07
tACS_alpha_	0.06 (0.02)	0.03	0.10
Effort_diff_	−0.06 (0.02)	−0.09	−0.03
Reward_high_	0.02 (0.02)	−0.01	0.05
tACS_theta_ × Effort_diff_	−0.00 (0.02)	−0.04	0.04
tACS_alpha_ × Effort_diff_	−0.03 (0.02)	−0.06	0.00
tACS_theta_ × Reward_high_	−0.04 (0.02)	−0.08	0.01
tACS_alpha_ × Reward_high_	−0.05 (0.02)	−0.09	−0.00
Reward_high_ × Effort_diff_	−0.00 (0.02)	−0.03	0.03
tACS_theta_ × Effort_diff_ × Reward_high_	−0.01 (0.02)	−0.01	0.07
tACS_alpha_ × Effort_diff_ × Reward_high_	0.03 (0.02)	−0.01	0.07

**Fig. 3:**
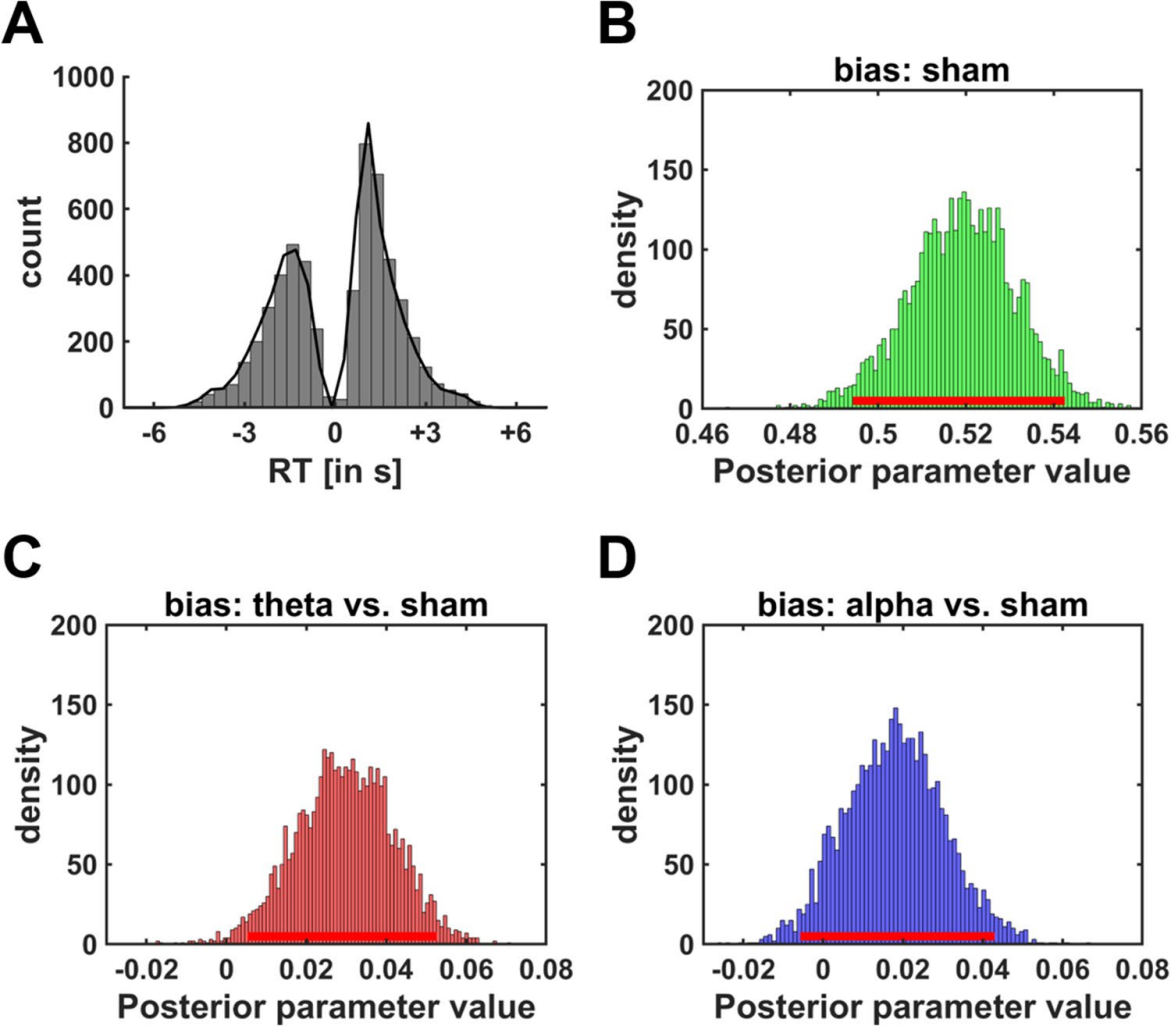
**Results of the hierarchical Bayesian drift diffusion model**. **a** Reaction time (RT) distributions in the effort-based decision task, with negative and positive RTs indicating choices of the low effort and high effort reward options, respectively. Black curves show simulated RT data based on individual parameter estimates. The overlap between observed (grey bars) and simulated RTs (black curves) suggests that our model provides a reasonable explanation for the empirical data. **b**-**d** Posterior distributions of (**b**) the starting bias parameter under sham as well as the impacts of (**c**) theta and (**d**) alpha tACS on the starting bias. Theta tACS significantly shifted the starting point of the evidence accumulation process towards the boundary of the high effort option. Red bars close to x-axis show 95% HDIs

From the perspective of process models, this finding could be explained by two different mechanisms. First, theta tACS could modulate the influence of benefits and costs on the speed of the evidence accumulation process (drift rate parameter). Second, instead of affecting evidence accumulation per se, midfrontal tACS might shift the starting point of the accumulation process toward the high effort option. These alternatives can be distinguished via drift diffusion modelling (DDM). In DDMs, the decision process is modelled as evidence accumulation process with a drift rate v (starting after the nondecision time t and from the starting point b) until the threshold a is reached. We computed four separate DDMs assuming that the drift rate of the accumulation process depends either on attribute-wise comparisons of reward magnitudes and N-back levels (DDM_attribute_) or on option-wise comparisons of discounted subjective reward values as given via hyperbolic, parabolic, or linear discount functions. DDM_attribute_ explained the data better than any other model, as indicated by a lower DIC (DIC_attribute_ = 15987; DIC_hyperbolic_ = 16270; DIC_parabolic_ = 19262; DIC_linear_ = 19532). Posterior predictive checks for DDM_attribute_ revealed strong overlap between simulated and empirically observed decision times, suggesting that the DDM parameters provide a reasonable explanation for the observed choices (Fig. 3a). As to be expected, larger differences in reward magnitudes biased evidence towards the high reward-high effort option, HDI_mean_ = 0.22, HDI_95%_ = [0.14; 0.31], whereas larger differences in effort requirements drove evidence accumulation towards the low effort option, HDI_mean_ = 0.12, HDI_95%_ = [0.02; 0.21]. Neither theta nor alpha tACS changed the influences of rewards or effort costs on evidence accumulation, as all HDI_95%_ included zero (Table 2). However, theta tACS significantly shifted the starting point of the accumulation process (bias parameter b) towards the high effort option, HDI_mean_ = 0.03, HDI_95%_ = [0.003; 0.05], whereas alpha tACS showed no significant effect, HDI_mean_ = 0.01, HDI_95%_ = [−0.01; 0.04] (Fig. 3b-d). We observed no significant stimulation effects on the decision threshold or on the non-decision time (Table 2). Note also that in the other three DDMs (although they explained the data worse than DDM_attribute_) theta tACS, relative to sham tACS, significantly increased the bias parameter. Thus, consistent with our model-free findings, midfrontal theta tACS increased the preference for high effort options via shifting the starting point of the evidence accumulation process.

**Table 2:** Results of the hierarchical Bayesian DDM (DDM_attribute_). We report parameter estimates under sham as well as the impact of tACS (tACS_theta_ and tACS_alpha_) relative to sham stimulation on DDM parameters. Standard errors of the mean of the posterior distributions are in brackets

Parameter	Regressor	Mean	2.5%	97.5%
Drift rate: β_reward_	Sham	0.22 (0.04)	0.14	0.31
	tACS_theta_	0.01 (0.03)	−0.05	0.08
	tACS_alpha_	0.02 (0.03)	−0.04	0.08
Drift rate: β_effort_	Sham	0.12 (0.05)	0.02	0.21
	tACS_theta_	0.04 (0.03)	−0.02	0.10
	tACS_alpha_	0.01 (0.03)	−0.05	0.08
Bias	Sham	0.52 (0.01)	0.50	0.55
	tACS_theta_	0.03 (0.01)	0.003	0.05
	tACS_alpha_	0.01 (0.01)	−0.01	0.04
Boundary	Sham	2.09 (0.08)	1.94	2.25
	tACS_theta_	0.02 (0.04)	−0.04	0.09
	tACS_alpha_	0.00 (0.03)	−0.07	0.08
Nondecision time	Sham	0.66 (0.04)	0.58	0.73
	tACS_theta_	0.01 (0.01)	−0.01	0.03
	tACS_alpha_	−0.00 (0.01)	−0.02	0.02

### No evidence for effects of midfrontal tACS on working memory performance

**Fig. 4:**
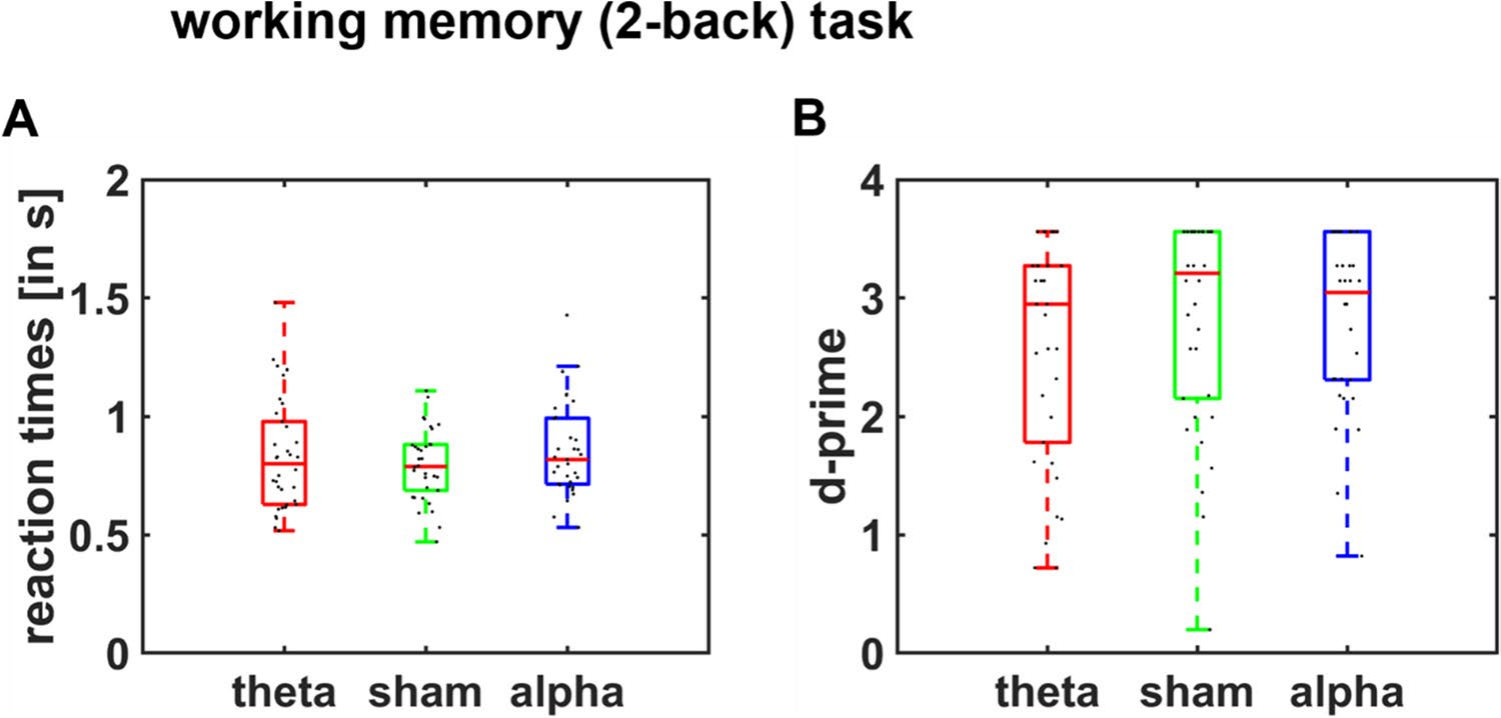
**tACS effects on the 2-back task.** We observed no significant stimulation effects on (**a**) reaction times or (**b**) d-prime (z-transformed hit rate minus false alarms) in the 2-back task as measures of working memory performance. Black dots indicate individual data points

Finally, we tested whether the stimulation effects on effort-based choice can be explained by changes in the capacity to exert mental effort. To control for this possibility, we assessed whether tACS affected performance in the 2-back working memory task. However, we observed no significant differences between stimulation conditions in reaction times, tACS_theta_: HDI_mean_ = 23.59, HDI_95%_ = [−51.08; 103.43], tACS_alpha_: HDI_mean_ = 47.80, HDI_95%_ = [−30.63; 123.45], or in sensitivity d’, tACS_theta_: HDI_mean_ = -0.16, HDI_95%_ = [−0.44; 0.11], tACS_alpha_: HDI_mean_ = −0.01, HDI_95%_ = [−0.30; 0.28] (Fig. 4). There was thus no evidence for tACS effects on working memory performance.

To control for the possibility that (albeit non-significant) tACS-induced differences in working memory performance led to the observed tACS effects on decision making, we re-computed MGLM-1 on subjective values in the decision task and controlled for working memory performance either via mean reaction times or sensitivity d’. The main effects of theta and alpha tACS remained significant independently of whether we controlled for reaction times (tACS_theta_: HDI_95%_ = [0.00; 0.07]; tACS_alpha_: HDI_95%_ = [0.02; 0.10]) or sensitivity d’ (tACS_theta_: HDI_95%_ = [0.00; 0.07]; tACS_alpha_: HDI_95%_ = [0.02; 0.10]). This does not support the possibility that tACS affected effort-based choice via changing the capacity to exert mental effort.

## Discussion

Previous research related decisions to engage in mental effort to a brain network including the dopaminergic midbrain (Westbrook et al., 2020) and prefrontal brain regions (Chong et al., 2017; Schmidt et al., 2012; Soutschek et al., 2018; Soutschek, Bagaini, et al., 2021a; Soutschek & Tobler, 2020; Vassena et al., 2017). However, less is known about the neural oscillations underlying the motivation to exert rewarded mental effort. Here, we show that midfrontal theta tACS causally increased the willingness to engage in goal-directed mental effort. Computational modelling with a process model of decision making provides insights into which subcomponent of the choice process was affected by theta tACS. While we observed no evidence for influences of tACS on the velocity of evidence accumulation (drift rate) or on the amount of evidence that is required for a decision (decision boundary), our findings suggest that midfrontal theta tACS shifted the starting point of the accumulation process toward the high reward-high effort option. Thus, midfrontal theta tACS did not change the sensitivity to reward values or effort costs but generally increased the propensity to choose high reward-high effort options. It seems unlikely that the stimulation effects on the starting bias can be explained by altered risk perception (assuming that higher effort levels are considered as riskier), because in this case the tACS effects should have depended on the difference in effort requirements between the low and high effort options. Moreover, we minimized potential influences of risk considerations by ensuring participants that they would obtain of the bonus as long as they work hard enough. It is further worth noting that performance in the working memory task remained unaffected by the stimulation, which speaks against the possibility that the impact of midfrontal tACS on decision making can be explained by changes in the capacity to exert mental effort. Thus, while we cannot formally rule out potential alternative interpretations (and we acknowledge that the observed tACS effects are relatively small), an increased willingness to engage in rewarded mental effort appears to be the best explanation for the current result pattern.

Note that in the model-free analysis also alpha tACS showed significant effects on effort-based decisions, though this effect was not significant in the DDM analysis. Alpha tACS was shown to induce theta oscillations (Moliadze et al., 2019), which might explain why theta and alpha tACS showed similar effects in the model-free analysis. However, given the role of alpha oscillations in top-down processes (Klimesch et al., 2007), it remains possible that alpha oscillations too are involved in effort-based decisions. This is in line with previous EEG evidence that both midfrontal alpha and theta oscillations are synchronized during feedback processing (Cohen et al., 2008). Although the effects of alpha tACS were not significant in the DDM analysis and thus appear to be less robust than the impact of theta tACS, the current data do not allow drawing any conclusions regarding the frequency-specificity of our findings. In other words, there is no evidence that theta oscillations are more strongly involved in effort-based decision making than alpha oscillations.

Midfrontal theta oscillations relate to the recruitment of cognitive control processes (Cavanagh et al., 2013; Cooper et al., 2019; Duprez et al., 2020; Pastötter et al., 2013; van Driel et al., 2015). The current findings suggest a reinterpretation of these results according to which midfrontal theta reflects an active decision making process which determines the strength of recruited cognitive control resources depending on whether the effort is “worth it.” This is consistent with recent theoretical frameworks understanding goal-directed mental effort as a cost-benefit weighing process (Kool & Botvinick, 2018; Shenhav et al., 2017). Our findings inform these accounts by suggesting that midfrontal theta tACS increases the motivation for rewarded effort not by changing the weights assigned to benefit or effort costs (which would increase the likelihood of both accepting rewards that are worth the associated effort demands and of rejecting rewards that are not worth it). Instead, theta tACS biases decisions towards accepting high reward-high effort options independently of the reward magnitudes and effort costs at stake by shifting the starting point of the evidence accumulation process closer to the decision boundary for high effort options. While the current findings seem consistent with the hypothesized role of midfrontal theta for mental effort, it is important to note that due to the lack of electrophysiological data it is not possible to decide whether the tACS effects on behavior are indeed driven by stimulation-induced entrainment of midfrontal theta oscillations.

Our findings have important implications for neural models of effort-based decision making. A large body of evidence ascribes the dACC a crucial role in weighing the costs of effort against the potential benefits (Bonnelle et al., 2016; Chong et al., 2017; Shenhav et al., 2013; Vassena et al., 2017). The dACC is hypothesized to top-down modulate striatal signals encoding the subjective value of effortful rewards options (Walton et al., 2009) and to recruit cognitive control processes in DLPFC (Domenech et al., 2018; Vassena et al., 2017; Vassena et al., 2019). So far, evidence in humans regarding dACC involvement in decision making has been only correlative in nature. Midfrontal theta oscillations are thought to be generated in dmPFC and dACC (Holroyd & Umemoto, 2016), such that it seems plausible to assume that the observed tACS effects can be explained by entrainment of theta oscillations in these regions. We note that besides dACC also the dmPFC was related to effort-based decision making (McGuire & Botvinick, 2010; Nagase et al., 2018). This interpretation needs to be taken with caution, however, given the low local specificity of tACS. Simulations of the current flow suggest that besides dmPFC/dACC also the DLPFC was affected by tACS in the current experiment, though the nonsignificant tACS effects on working memory performance (given that working memory processes were linked to theta oscillations in DLPFC (Alekseichuk et al., 2017; Röhner et al., 2018)) do not support the possibility that the observed results are caused by stimulation effects on DLPFC. Nevertheless, we refrain from making strong claims regarding the localization of the tACS effects and instead interpret our findings more carefully as evidence for an impact of midfrontal tACS (irrespective of the precise neural generators) on effort-based choice.

It is worth noting some methodological limitations of the current investigation. First, as in most tDCS studies, experimenters were not blinded to the current tACS condition, which could lead to experimenter demand effects. However, it seems rather unlikely that such demand effects resulted in the observed differences between active and sham tACS given that experimenters did not interact with participants during task performance under tACS (note that participants underwent all three tACS conditions within one single experimental session). We also did not directly assess whether blinding of participants was successful as we did not ask participants whether they currently received sham or active tACS in a given miniblock. However, tACS conditions did not significantly differ with regard to tACS-induced discomfort or flickering, suggesting that active and sham tACS were virtually indistinguishable to participants. As we moreover statistically controlled for such tACS-induced irritations in our statistical models, it seems unlikely that peripheral effects drove the observed stimulation effects on decision making.

Taken together, our results provide insights into the neural mechanisms underlying the willingness to engage in rewarded mental effort. Deficits in the motivation to exert effort belong to the core deficits of several psychiatric disorders, including depression and schizophrenia (Hartmann et al., 2015; Kreis et al., 2020; Patzelt et al., 2019; Rock et al., 2014; Scheurich et al., 2008). By providing a causal link between midfrontal tACS and the willingness to engage in effort, our findings suggest that these motivational deficits could be treated by neural interventions targeting dmPFC.

## Data Availability

The data that support the findings of this study will be available on the Open Science Framework (https://osf.io/a2bhy/).

## Code availability

Data analysis code will be available on the Open Science Framework (https://osf.io/a2bhy/).
